# Psychiatric pharmaceutical care service across Malaysian hospitals: results from a cross-sectional study

**DOI:** 10.1186/s12913-022-07681-4

**Published:** 2022-03-09

**Authors:** Aya Ahmed Abousheishaa, Ahmad Hatim Sulaiman, Hasniza Zaman Huri, Siti Fatimah Binti Kamis, Hafizah Hamidi, Wei Chern Ang, Zainol Akbar bin Zainal, Noorasyikin Shamsuddin, Ng Chong Guan

**Affiliations:** 1 Department of Psychological Medicine, Faculty of Medicine, University of Malaya, 50603 Kuala Lumpur, Malaysia; 2 Faculty of Pharmacy, University of Malaya, 50603 Kuala Lumpur, Malaysia; 3 Hospital Sultan Ismail, 81100 Johor Bahru, Johor Malaysia; 4 Hospital Permai, 81200 Johor Bahru, Johor Malaysia; 5 Hospital Tuanku Fauziah, 01000 Kangar, Perlis Malaysia; 6 Faculty of Pharmacy, University of Cyberjaya, 63000 Cyberjaya, Selangor Malaysia

**Keywords:** Psychiatric pharmaceutical services, Pharmacy services, Conventional services, Hospital pharmacy, Pharmacist, Barriers, Mental health

## Abstract

**Background:**

Psychiatric pharmaceutical care is the provision of pharmaceutical care services to patients with psychiatric related illnesses or disorders. Several studies have demonstrated the positive influence psychiatric pharmaceutical care on patients’ clinical, humanistic and economic outcomes. This study aimed to examine the extent of psychiatric pharmaceutical care practice in a convenience sample of Malaysian government hospitals and the barriers to the provision of these services.

**Methods:**

An anonymous cross-sectional survey of registered pharmacists working at a convenience sample of government hospitals in Malaysia was undertaken from September 2019 to June 2020.

**Results:**

Pharmacists frequently ensured the appropriateness of the dose (55%), dosage form (47%) and dosing schedule (48%) of the dispensed medications. Most pharmacists infrequently worked with patients and healthcare professionals to develop a pharmacotherapeutic regimen and a corresponding monitoring plan (28%). There was no statistically significant difference in the provision of pharmaceutical care services with respect to gender, age, years of practice, and professional board certification. However, the services offered were influenced by the respondent’s education and pharmacy setting. The obstacles perceived by pharmacists included lack of time (89%), shortage of pharmacy staff (87%), the patients’ inability to comprehend medical information (85%), insufficient demand and acceptance by patients (82%), the lack of official policies and standardised practice protocols (78%), inaccessibility to the patients’ medical records (77%) and the lack of structured communication channels between pharmacists and physicians (75%), the pharmacists lack of knowledge/skills and confidence (78%) and insufficient recognition from physicians to the pharmacists’ skills (76%).

**Conclusions:**

This is the first study to explore the extent and barriers of psychiatric pharmaceutical care in Malaysian hospitals; it highlighted the need for mobilising pharmacists to expand these services.

## Background

The past few decades witnessed a global shift in the disease burden from communicable to non-communicable diseases [1]. Currently, non-communicable diseases (NCDs) constitute the majority of premature deaths in most parts of the world, including low and middle-income countries [1–3]. The most recognised of these diseases are cancer, cardiovascular diseases, chronic obstructive pulmonary diseases, and diabetes [4]. However, more than half of the global NCD burden arises from other diseases, including mental health and addictive disorders [5]. In 2016, more than a billion people were affected with mental health or addictive disorders, i.e., more than 16% of the world’s population [6]. The same year observed a loss of 162.5 million disability-adjusted life years (DALYs) due to mental health or addictive disorders [6]. The disabling nature of mental health and addictive disorders is echoed in the DALYs, as it captures both the number of years of life lost due to premature mortality combined with the years lost due to disability [7].

The pharmacological management of mental health and addictive disorders is widely recognised. However, medication-related morbidity and mortality, particularly among this patient population, are on the rise [8, 9] and has an enormous cost that potentially surpasses the drugs’ costs [10]. Drug-related problems (DRPs) involve an event or circumstance related to drug therapy that actually or potentially interferes with desired health outcomes. It can occur at any level of the medicine use cycle, including prescribing, dispensing, and using the medication [11]. The emergence of pharmaceutical care in 1990 as a philosophy of practice offered promising prospects to implementing and optimising pharmacotherapy [12]. It introduced a shift in the profession from a drug-oriented practice to a patient-centred approach. The process entails the pharmacist’s involvement in identifying, resolving, and preventing drug-related problems to achieve optimal outcomes that enhance patients` quality of life [13].

“Psychiatric pharmacy” in countries like the United States and the United Kingdom is a recognised specialty whereby pharmacists provide pharmaceutical care to patients with mental health disorders [14, 15]. Several studies have exhibited the positive influence of psychiatric pharmaceutical care on patients’ clinical, humanistic and economic outcomes [16]. Furthermore, investigations of varying nature featured in a systematic review of the impact of pharmacist interventions on mental health demonstrated an enhancement in the safety and efficacy of psychotropic drug use [17]. Despite its advantages, the provision of pharmaceutical care services has not yet been widely adopted into practice by many pharmacists. Reasons for this vary by the country however potential barriers include pharmacists’ limited knowledge of mental health disorders, misconception related to the cognitive ability of mental health patients, lack of access to the patients’ medical records, and physicians’ perception of the pharmacists’ roles [18].

To date, no information is available on the level and scope of psychiatric pharmaceutical care in Malaysian hospitals. Furthermore, factors that hinder pharmacists from the routine practice of pharmaceutical care are yet to be studied. Once identified, these factors can be targeted to design effective intervention programs that can be employed to foster wider adoption of pharmacy practice in mental health.

This study aims to examine the extent of psychiatric pharmaceutical care practice in a convenience sample of Malaysian government hospitals and the barriers that impede pharmacists from providing these services. Furthermore, it will examine the impact of pharmacists’ demographic and practice characteristics on the level of pharmacy practice, to determine factors that are more likely to influence patterns in service provision.

## Materials and methods

### Study design, setting and participants

An anonymous cross-sectional survey of registered pharmacists working at a convenience sample of government hospitals in Malaysia was undertaken from September 2019 to June 2020. Onsite co-investigators recruited pharmacists based on the following eligibility criteria (1) registered pharmacists (2) experienced in the provision of pharmaceutical care to patients with psychiatric-related illnesses or disorders.

### Survey instrument

A self-administered questionnaire was developed in English following a thorough literature review. It was founded on the American Society of Hospital Pharmacists (ASHP) guidelines on standardised methods for pharmaceutical care [19], Society of Hospital Pharmacists of Australia (SHPA) standards of practice for mental health pharmacy [20] and Pharmaceutical Care Network Europe (PCNE) classification for drug-related problems V8.02 [21]. The official language in Malaysia is Bahasa Melayu; however, previous studies have indicated the feasibility of using English language questionnaires to collect data from practicing pharmacists [22]. Content validation of the questionnaire was achieved through expert judgment by seven faculty members and researchers in the field of pharmacy practice across different universities in Malaysia. Furthermore, it was tested for face validity (i.e., comprehensibility, applicability, and acceptability) among a convenience sample of 11 pharmacists practicing at the hospitals in which the research was carried out. These pharmacists did not take part in the actual survey. The results indicated that some questions were difficult to comprehend or contextually irrelevant. Subsequently, these questions were paraphrased, and the questionnaire was finalised. The final version of the questionnaire had three separate sections that could be filled in 15-20 minutes.

The questionnaire commenced with an introduction outlining the objectives and importance of the research, followed by the definition of psychiatric pharmaceutical care and eligibility criteria for participation. It also included an ethical statement regarding participant consent and data anonymisation. The first set of questions captured the sociodemographic information of the responding pharmacists and the characteristics of their pharmacy practice. In the second section, pharmacists were asked about the extent of their pharmaceutical care services to psychiatric patients via a five-point Likert-type scale, i.e., 1= Never, 2 = Rarely, 3 = Sometimes, 4 = Often and 5 = Always. It had statements to characterise the assessment of drug-related problems, pharmacist interventions, provision of pharmaceutical care supporting materials to patients and caregivers, and the use of information resources to guide the pharmaceutical care process. This aimed at documenting the type and frequency of the pharmaceutical care services actually delivered by the pharmacists. The last section of the questionnaire addressed the challenges that hinder the provision of pharmaceutical care to psychiatric patients; it had a list of items categorised as patient, pharmacist and health system-related factors. The pharmacists were requested to express their perception of these barriers on a 5-point Likert scale from 1= Strongly disagree to 5= Strongly agree. The participants had the option to write any additional barriers that were not listed to better represent the challenges.

### Sample size

The total number of pharmacists practising in the sampled hospitals at the time of the study was 560. Raosoft sample size calculator was used to estimate the sample size, utilizing a 50% response distribution [23].

### Survey implementation

The data was collected using google forms, the internet-based survey tool. Pharmacists appointed as on-site co-investigators at the included hospitals utilised the internal staff database to email the survey link to all pharmacists working in different settings within the hospital. Eligible and consenting pharmacists could complete the survey. Reminders via email were sent at two-week intervals to ensure the completion of the questionnaire.

### Data analysis

Data analysis was carried out using IBM Statistical Package of Social Sciences (SPSS) Version 25. Invalid and incomplete data was assumed missing. Pairwise deletion technique was used to handle the missing data; this seemed to be an appropriate mechanism since there were few missing observations. Missing data analysis revealed that the data set had less than 0.5% missing values and non of the variables had more than 2.5% missing values. Frequencies, percentages, means and standard deviations were used to summarise the sociodemographic and practice characteristics of the pharmacists. Frequencies and percentages were used to determine the frequency of assessment of drug-related problems, pharmacist interventions, provision of pharmaceutical care supporting material to patients and caregivers, and the use of supporting material and educational resources. Furthermore, median scores of these data were also reported. The pharmacists’ perceived barriers to the provision of pharmaceutical care were illustrated using a bar chart; the scores for “strongly disagree” and “disagree” were added to indicate their level of disagreement with the provided statements, and the scores for “strongly agree” and “agree” were similarly transformed to reflect their extent of agreement.

To determine patterns in service provision, contingency tables were created for the pharmacists’ assessment of drug-related problems, pharmacists’ interventions, and demographics, including age, gender, education, years of practice, and practice setting. The interrelationship among cross-tabulated data was tested using the chi-square test of independence and fisher-freeman-halton exact test. Since the chi-square test relies on applying approximation method, the fisher's exact test was used when more than 20% of cells had expected frequencies < 5. A *p*-value of less than 0.05 was considered significant.

## Results

All the pharmacists practising in the sampled hospitals at the time of the study were surveyed. One hundred and seventy-six responses were obtained during the seven-month collection period. Eight questionnaires had no responses and were excluded from the study. The remaining one hundred and sixty-eight responses were included in the data analysis (response rate = 30%).

The sociodemographic characteristics and pharmacy-related information of the respondents are summarised in Table 1. The mean age of the respondents was approximately 30 years, with a higher female prevalence (*n*= 138). The pharmacists have been practising for an average of six years with two years of experience in mental health. Most respondents had a bachelor’s degree in pharmacy (82.7 %) and no professional board certification (97.6 %). Nearly half of the pharmacists practised in outpatient pharmacies (42.3%).

**Table 1:** Sociodemographic and practice characteristics of the respondents

Characteristic	Frequency (%)
Age (*N* = 168) mean (SD)	30.58 (3.99)
Gender (*N*=167)	
Male	29 (17.3 %)
Female	138 (82.1 %)
Ethnicity (*N* = 168)	
Malay	88 (52.4 %)
Chinese	57 (33.9 %)
Indian	20 (11.9 %)
Other	3 (1.8 %)
Pharmacy education (*N* = 168)	
Bachelor’s degree	139 (82.7 %)
Other degrees (Pharm D, MSc, M Pharm)	29 (17.2 %)
Professional Board Certification (*N* = 168)	
No	164 (97.6 %)
Yes	4 (2.4 %)
Professional training on the management of patients with psychiatric-related illnesses/ disorders (*N* = 168)	
No	138 (82.1 %)
Yes	30 (17.9 %)
Years of practicing pharmacy (*N* =167)	6.06 (3.92)
Years of practice in the field of psychiatric related illnesses/ disorders (*N* = 164)	1.90 (2.79)
Time since pharmaceutical care services was last provided (*N* = 166)	
< 1month	72 (42.9 %)
1 - 2 months	31 (18.5 %)
2 - 6 months	22 (13.1 %)
6 - 12 months	21 (12.5 %)
1 - 5 years	18 (10.7 %)
> 5 years	2 (1.2 %)
Pharmacist Position (*N* =167)	
Clinical Pharmacist	17 (10.1 %)
Medication Therapy Adherence Clinic	7 (4.2 %)
Ward Pharmacist	34 (20.2 %)
Drug Information Centre	8 (4.8 %)
Outpatient Pharmacist	71 (42.3 %)
Inpatient Pharmacy	21 (12.5 %)
Other	9 (5.4 %)
Hospital (*N*=168)	
Hospital Bahagia Ulu Kinta	25 (14.9 %)
Hospital Banting	7 (4.2 %)
Hospital Mesra Bukit	2 (1.2 %)
Hospital Permai Johor Bahru	21 (12.5 %)
Hospital Queen Elizabeth	9 (5.4 %)
Hospital Sultan Ismail	37 (22.0 %)
Hospital Sultanah Aminah	6 (3.6 %)
Hospital Sungai Buloh	26 (15.5 %)
Hospital Tuanku Fauziah	24 (14.3 %)
University of Malaya Medical Centre	9 (5.4 %)
Pejabat Kesihatan Daerah Kuantan	2 (1.2 %)

Tables 2 and 3 summarise the pharmacists’ reported assessment of drug therapy problems and performance of pharmaceutical care interventions at different levels of the medication use process. While the pharmacists examined patient prescriptions for some drug therapy problems, around 50% of the respondents did not allocate sufficient time attending to essential aspects of the pharmaceutical care process. The pharmacists often or always ensured the appropriateness of the dose (55%), dosage form (47%) and dosing schedule (48%) of the dispensed medications. They evaluated the patients for adherence to therapy (60%), adverse drug reactions (45%), assessed their information needs (60%) and engaged in patient education and counselling (63%). However, other services were performed to a much lesser extent, including monitoring patients for therapeutic drug efficacy (14%) and drug toxicity (23%).

**Table 2:** Respondents` assessment of prescriptions for drug therapy problems

Service	Never	Rarely	Sometimes	Often	Always	Total	Median
	*N* (%)	*N* (%)	*N* (%)	*N* (%)	*N* (%)		
**Drug Prescribing**							
Inappropriate drug	14 (8.3%)	49 (29.2 %)	48 (28.6 %)	31 (18.5 %)	26 (15.5 %)	168	3
No indication for drug	21(12.7%)	43 (25.9 %)	46 (27.7 %)	27 (16.3 %)	29 (17.5 %)	168	3
No drug treatment inspite indication	34 (20.4%)	50 (29.9 %)	41 (24.6 %)	21 (12.6 %)	21 (12.6 %)	168	2
Drug contra-indicated	17 (10.1%)	35 (20.8 %)	41 (24.4 %)	50 (29.8 %)	25 (14.9 %)	168	3
Inappropriate combination of drugs or herbals	15 (9.0 %)	34 (20.4 %)	52 (31.1 %)	43 (25.7 %)	23 (13.8 %)	168	3
Inappropriate drug dose	8 (4.8 %)	21 (12.5 %)	46 (27.4 %)	38 (22.6 %)	55 (32.7 %)	168	4
Inappropriate dosage form	17 (10.1 %)	37 (22.0 %)	34 (20.2 %)	38 (22.6 %)	42 (25.0 %)	168	3
Inappropriate dosage schedule	15 (8.9 %)	31 (18.5 %)	40 (23.8 %)	45 (26.8 %)	37 (22.0 %)	168	3
vInappropriate duration of therapy	14 (8.4 %)	32 (19.3 %)	50 (30.1 %)	35 (21.1 %)	35 (21.1 %)	168	3
**Drug Dispensing**							
Drug unavailable	4 (2.4 %)	30 (18.1 %)	72 (43.4 %)	28 (16.9 %)	32 (19.3 %)	168	3
Incorrect drug/ strength dispensed	11 (6.6 %)	48 (28.7 %)	48 (28.7 %)	22 (13.2 %)	38 (22.8 %)	168	3
Patient/ caregiver information needs	5 (3.0 %)	30 (18.1 %)	50 (30.1 %)	40 (24.1 %)	41 (24.7 %)	168	3
Inaccurate patient medication list	7 (4.2 %)	50 (30.3 %)	49 (29.7 %)	27 (16.4 %)	32 (19.4 %)	168	3
**Drug Use**							
Complicated therapeutic regimen	9 (5.4 %)	48 (28.6 %)	60 (35.7 %)	33 (19.6 %)	18 (10.7 %)	168	3
Incorrect drug administered/ used	15 (9.0 %)	54 (32.3 %)	41 (24.6 %)	30 (18.0 %)	27 (16.2 %)	168	3
Incorrect route of administration	31 (18.6 %)	44 (26.3 %)	39 (23.4 %)	26 (15.6 %)	27 (16.2 %)	168	3
Incorrect dosing interval	14 (8.3 %)	50 (29.8 %)	46 (27.4 %)	25 (14.9 %)	33 (19.6 %)	168	3
Incorrect duration of therapy	14 (8.4 %)	51 (30.5 %)	50 (29.9 %)	27 (16.2 %)	25 (15.0 %)	168	3
Drug abuse	25 (14.9 %)	48 (28.6 %)	53 (31.5 %)	27 (16.1 %)	15 (8.9 %)	168	3
Unnecessary drug use	15 (8.9 %)	58 (34.5 %)	54 (32.1 %)	23 (13.7 %)	18 (10.7 %)	168	3
Expired drug use	37 (22.3 %)	43 (25.9 %)	35 (21.1 %)	25 (15.1 %)	26 (15.7 %)	168	3
Incorrect drug storage	23 (13.7 %)	42 (25.0 %)	48 (28.6 %)	32 (19.0 %)	23 (13.7 %)	168	3
Non-compliance to therapy	8 (4.8 %)	13 (7.8 %)	46 (27.5 %)	55 (32.9 %)	45 (26.9 %)	168	4
**Other**							
Drug therapy ineffectiveness (ex. therapeutic resistance)	12 (7.1 %)	50 (29.8 %)	82 (48.8 %)	15 (8.9 %)	9 (5.4 %)	168	3
Adverse drug reaction	10 (6.0 %)	20 (12.0 %)	62 (37.1 %)	48 (28.7 %)	27 (16.2 %)	168	3
Drug toxicity	16 (9.5 %)	16 (9.5 %)	54 (32.1 %)	26 (15.5 %)	13 (7.7 %)	168	3

**Table 3:** Respondents` performance of pharmaceutical care interventions

Interventions	Never	Rarely	Sometimes	Often	Always	Total	Median
	*N* (%)	*N* (%)	*N* (%)	*N* (%)	*N* (%)		
Develop a pharmacotherapeutic regimen and corresponding monitoring plan with the patient and other healthcare professionals	26 (15.6 %)	40 (24.0 %)	54 (32.3 %)	30 (18.0 %)	17 (10.2 %)	168	3
Recommend changes to pharmacotherapeutic regimen and corresponding monitoring plan with the patient and other health care professionals	17 (10.2 %)	35 (21.1 %)	58 (34.9 %)	37 (22.3 %)	19 (11.4 %)	168	3
Patient/ caregiver counselling and education	14 (8.5 %)	15 (9.1 %)	32 (19.5 %)	51 (31.1 %)	52 (31.7 %)	168	4

Moreover, the prescriptions were never or seldom assessed for (Table 2) untreated medical indications (50.3%), incorrectly indicated (37.5%), dispensed (35.3%), or administered medications (41.3%) and drug misuse or abuse (43.5%). Likewise, the patients were rarely monitored for the use of expired medications (48.2%). Most pharmacists infrequently worked with patients and healthcare professionals to develop a pharmacotherapeutic regimen and a corresponding monitoring plan (28%); in fact, recommendations to change the existing therapeutic regimen and follow-up scheme were as low as 34%.

There was no statistically significant difference in the provision of psychiatric pharmaceutical care services with respect to gender, age, years of practice, and professional board certification. However, the services offered seemed to be influenced by the respondent’s education. Pharmacists with a bachelor’s degree (Table 4) were more likely to engage in patient education and counselling (*p* = 0.005) compared to pharmacists with other educational qualifications. Likewise, the extent of pharmaceutical care varied by the practice setting (Table 5). Outpatient pharmacists followed by ward pharmacists appear to evaluate the appropriateness of the dosage form prescribed (*p* = 0.03), the correctness of the drug/strength dispensed (*p* = 0.003), the accuracy of patient medication list (*p* = 0.01), the correctness of the drug administered (*p* = 0.008), the use of expired medication (*p* = 0.017) and the potential for adverse drug reactions (*p* = 0.048) to a greater extent than other practice settings; Furthermore they engaged more in developing (*p* = 0.033) and amending (*p* = 0.033) pharmacotherapeutic regimens and the corresponding monitoring plans with patients and other healthcare professionals.

**Table 4:** Effect of pharmacists’ education on the provision of pharmaceutical care services

Service	Education level	Never *N* (%)	Rarely *N* (%)	Sometimes *N* (%)	Often *N* (%)	Always *N* (%)	Total	Significance level (2 sided)
Provide patient education and counselling	Bachelor’s degree	11 (8 %)	10 (7 %)	24 (17 %)	45 (33 %)	46 (33 %)	136	0.005
	Pharm D	3 (17 %)	1(5 %)	8 (47 %)	3 (17 %)	2 (11 %)	17	
	Master’s degree	0 (0 %)	3 (30 %)	0 (0 %)	3 (30 %)	4 (40 %)	10	
	M Pharm	0 (0 %)	1 (100 %)	0 (0 %)	0 (0 %)	0 (0 %)	1

**Table 5:** Impact of the pharmacy setting on the provision of pharmaceutical care services

Service	Pharmacy setting	Never *N* (%)	Rarely *N* (%)	Sometimes *N* (%)	Often *N* (%)	Always *N* (%)	Total	Significance level (2 sided)
Assess appropriateness of the dosage form prescribed	Clinical Pharmacist	3 (17 %)	0 (0%)	3 (17 %)	3 (17 %)	8 (47%)	17	0.03
	Medication Therapy Adherence Clinic	1 (14%)	1 (14%)	1 (14%)	3 (43%)	1 (14%)	7	
	Ward Pharmacist	0 (0%)	14 (41%)	2 (6%)	11 (32%)	7 (20%)	34	
	Drug Information Centre	1 (12%)	1 (12%)	2 (25%)	1 (12%)	3 (38%)	8	
	Outpatient Pharmacist	9 (13%)	16 (22%)	17 (24%)	13 (18%)	16 (22%)	71	
	Inpatient Pharmacy	2 (9%)	2 (9%)	7 (33%)	4 (19%)	6 (29%)	21	
	Other	1 (11%)	2 (22%)	2 (22%)	3 (33%)	1 (11%)	9	
Assess correctness of drug/strength dispensed	Clinical Pharmacist	3 (17 %)	0 (0 %)	2 (11%)	6 (35 %)	6 (35 %)	17	0.003
	Medication Therapy Adherence Clinic	0 (0%)	4 (57 %)	2 (28 %)	0 (0 %)	1 (14 %)	7	
	Ward Pharmacist	2 (5 %)	13 (38 %)	6 (17 %)	4 (11 %)	9 (26 %)	34	
	Drug Information Centre	0 (0 %)	2 (25 %)	1 (12 %)	1 (12 %)	4 (50 %)	8	
	Outpatient Pharmacist	3 (4 %)	24 (34 %)	26 (37 %)	7 (10 %)	10 (14 %)	70	
	Inpatient Pharmacy	3 (14 %)	1 (4 %)	7 (33 %)	3 (14 %)	7 (33 %)	21	
	Other	0 (0 %)	3 (33 %)	4 (44 %)	1 (11 %)	1 (11 %)	9	
Assess accuracy of the medication list	Clinical Pharmacist	1 (5 %)	2 (11 %)	3 (17 %)	4 (23 %)	7 (41 %)	17	0.01
	Medication Therapy Adherence Clinic	1 (14 %)	2 (28 %)	1 (14 %)	2 (28 %)	1 (14 %)	7	
	Ward Pharmacist	3 (8 %)	14 (41 %)	7 (20 %)	5 (14 %)	5 (14 %)	34	
	Drug Information Centre	1 (12 %)	0 (0 %)	2 (25 %)	1 (12 %)	4 (50 %)	8	
	Outpatient Pharmacist	1 (1 %)	25 (35 %)	26 (37 %)	11 (15 %)	7 (10 %)	70	
	Inpatient Pharmacy	0 (0 %)	3 (15 %)	5 (26 %)	4 (21 %)	7 (36 %)	19	
	Other	0 (0 %)	3 (33 %)	5 (55 %)	0 (0 %)	1 (11 %)	9	
Assess correctness of the drug administered	Clinical Pharmacist	2 (11 %)	0 (0 %)	5 (29 %)	8 (47 %)	2 (11%)	17	0.008
	Medication Therapy Adherence Clinic	2 (28 %)	2 (28 %)	2 (28 %)	0 (0 %)	1 (14 %)	7	
	Ward Pharmacist	2 (5 %)	19 (55 %)	6 (17 %)	3 (8 %)	4 (11 %)	34	
	Drug Information Centre	1 (12 %)	2 (25 %)	0 (0 %)	1 (12 %)	4 (50 %)	8	
	Outpatient Pharmacist	8 (11 %)	21 (30 %)	20 (28 %)	11 (15 %)	10 (14 %)	70	
	Inpatient Pharmacy	0 (0 %)	5 (23 %)	6 (28 %)	5 (23 %)	5 (23 %)	21	
	Other	0 (0 %)	4 (44 %)	2 (22 %)	2 (22 %)	1 (11 %)	9	
Assess expired medication use	Clinical Pharmacist	4 (23 %)	4 (23 %)	1 (5 %)	3 (17 %)	5 (29 %)	17	0.017
	Medication Therapy Adherence Clinic	4 (57 %)	0 (0 %)	3 (42 %)	0 (0 %)	0 (0 %)	7	
	Ward Pharmacist	13 (38 %)	9 (26 %)	3 (8 %)	7 (20 %)	2 (5 %)	34	
	Drug Information Centre	1 (12 %)	1 (12 %)	2 (25 %)	2 (25 %)	2 (25 %)	8	
	Outpatient Pharmacist	12 (17 %)	22 (31 %)	18 (26 %)	10 (14 %)	7 (10 %)	69	
	Inpatient Pharmacy	2 (9 %)	3 (14 %)	6 (28 %)	2 (9 %)	8 (38 %)	21	
	Other	1 (11 %)	3 (33 %)	2 (22 %)	1 (11 %)	2 (22 %)	9	
Assess adverse drug reactions	Clinical Pharmacist	0 (0 %)	1 (5 %)	5 (29 %)	6 (35 %)	5 (29 %)	17	0.033
	Medication Therapy Adherence Clinic	1 (14 %)	0 (0 %)	0 (0 %)	4 (57 %)	2 (28 %)	7	
	Ward Pharmacist	2 (5 %)	6 (17 %)	10 (29 %)	13 (38 %)	3 (8 %)	34	
	Drug Information Centre	1 (12 %)	0 (0 %)	3 (37 %)	0 (0 %)	4 (50 %)	8	
	Outpatient Pharmacist	6 (8 %)	10 (14 %)	33 (46 %)	13 (18 %)	9 (12 %)	71	
	Inpatient Pharmacy	0 (0 %)	2 (9 %)	8 (38 %)	7 (33 %)	4 (19 %)	21	
	Other	0 (0 %)	1 (12 %)	2 (25 %)	5 (62 %)	0 (0 %)	8	
Develop a pharmacotherapeutic regimen and corresponding monitoring plan with the patient and other healthcare professionals	Clinical Pharmacist	1 (5 %)	2 (11 %)	3 (17 %)	4 (23 %)	7 (41 %)	17	0.033
	Medication Therapy Adherence Clinic	1 (14 %)	1 (14 %)	3 (42 %)	2 (28 %)	0 (0 %)	7	
	Ward Pharmacist	5 (14 %)	6 (17 %)	8 (23 %)	11 (32 %)	4 (11%)	34	
	Drug Information Centre	2 (25 %)	0 (0 %)	5 (62 %)	1 (12 %)	0 (0 %)	8	
	Outpatient Pharmacist	14 (20 %)	22 (31 %)	23 (32 %)	8 (11 %)	3 (4 %)	70	
	Inpatient Pharmacy	2 (9 %)	6 (28 %)	9 (42 %)	3 (14 %)	1 (4 %)	21	
	Other	1 (11 %)	2 (22 %)	3 (33 %)	1 (11 %)	2 (22 %)	9	
Recommend changes to pharmacotherapeutic regimen and corresponding monitoring plan with the patient and other health care professionals	Clinical Pharmacist	1 (5 %)	2 (11 %)	3 (17 %)	2 (11 %)	9 (52 %)	17	0.033
	Medication Therapy Adherence Clinic	1 (14 %)	0 (0 %)	4 (57 %)	2 (28 %)	0 (0 %)	7	
	Ward Pharmacist	4 (11 %)	6 (17 %)	6 (17 %)	14 (41 %)	4 (11 %)	34	
	Drug Information Centre	0 (0 %)	3 (37 %)	4 (50 %)	1 (12 %)	0 (0 %)	8	
	Outpatient Pharmacist	8 (11 %)	15 (21 %)	29 (42 %)	14 (20 %)	3 (4 %)	69	
	Inpatient Pharmacy	2 (9 %)	7 (33 %)	9 (42 %)	2 (9 %)	1 (4 %)	21	
	Other	1 (11 %)	1 (11 %)	3 (33 %)	2 (22 %)	2 (22 %)	9	

The use of pharmaceutical care supporting materials was not highly prevalent among most respondents (Table 6). Fewer than half of the pharmacists always or often provided patients with their medication list/ dosage schedules or auxiliary labels. Furthermore, less than one-third of the patients were given any information leaflets/written or printed educational material. Likewise, around 38 % of the pharmacists never or rarely supplied their patients with any adherence support tools.

**Table 6:** Respondents` provision of pharmaceutical care supporting materials to patient and caregiver

Supporting material	Never	Rarely	Sometimes	Often	Always	Total	Median
	*N* (%)	*N* (%)	*N* (%)	*N* (%)	*N* (%)		
Medication list/ dosage schedule	17 (10.1 %)	30 (17.9 %)	60 (35.7 %)	38 (22.6 %)	23 (13.7 %)	168	3
Patient information leaflet/ written or printed educational material	19 (11.3 %)	40 (23.8 %)	63 (37.5 %)	31 (18.5 %)	15 (8.9 %)	168	3
Auxillary labels	18 (10.7 %)	30 (17.9 %)	60 (35.7 %)	37 (22.0 %)	23 (13.7 %)	168	3
Adherence aid tools	25 (14.9 %)	39 (23.2 %)	67 (39.9 %)	27 (16.1 %)	10 (6.0 %)	168	3

Table 7 demonstrates that most pharmacists always rely on online drug information resources to guide the pharmaceutical care process (45.8 %). They seem to use treatment guidelines, protocols, algorithms and textbooks to a lesser degree, which indicates a potential deficiency in access or appropriateness of these information sources.

**Table 7:** Respondents` use of information sources to guide the pharmaceutical care process

Information sources	Never	Rarely	Sometimes	Often	Always	Total	Median
	*N* (%)	*N* (%)	*N* (%)	*N* (%)	*N* (%)		
Treatment guidelines, protocols, algorithms	3 (1.8 %)	11 (6.5 %)	28 (16.7 %)	63 (37.5 %)	63 (37.5 %)	168	4
Online drug information resources	3 (1.8 %)	6 (3.6 %)	23 (13.7 %)	59 (35.1 %)	77 (45.8 %)	168	4
Text book drug information sources	9 (5.4 %)	23 (13.8 %)	47 (28.1 %)	42 (25.1 %)	46 (27.5 %)	168	4

**Fig. 1:**
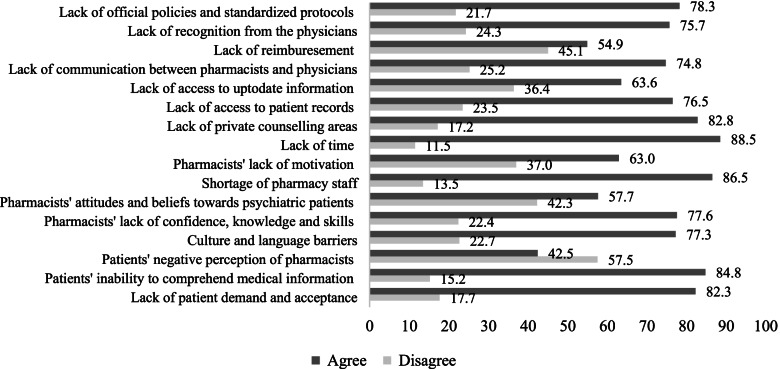
Perceived barriers to pharmaceutical care provision; data presented in percentages

The obstacles perceived by pharmacists to the provision of pharmaceutical care to patients with psychiatric related illnesses or disorders are illustrated in Fig. 1. Most pharmacists agreed that the lack of time (89%) and shortage of pharmacy staff (87%) impeded them from expanding the scope of pharmaceutical care services. Furthermore, the patients’ inability to comprehend medical information (85%), followed by their insufficient demand and acceptance (82%) of the pharmacists’ services, were commonly reported barriers. Health system-related factors, including the lack of official policies and standardised practice protocols (78%), inaccessibility to the patients’ medical records (77%) and the lack of structured communication channels between pharmacists and physicians (75%), were perceived to hinder the practice of pharmaceutical care. Another significant barrier accounted for by 78% of the pharmacists was their lack of knowledge/skills and confidence to provide pharmaceutical care to patients with psychiatric illnesses. Likewise, the insufficient recognition from physicians to the pharmacists’ skills hindered 76% of the pharmacists from providing appropriate pharmaceutical care.

## Discussion

The findings of this study deepen our understanding of psychiatric pharmaceutical care offered within government hospitals in Malaysia. It shows that pharmacists offer a wide range of services across different settings, although the extent and breadth of these services could be enhanced. Around half of the respondents often assess prescriptions for a limited range of potential medication-related problems, including adverse drug reactions. Moreover, monitoring patients for therapeutic efficacy, drug toxicity and medication misuse and abuse were seldom done. This is unfortunate, especially that patients with psychiatric related illnesses or disorders are at risk of developing drug therapy problems [24–26]. There is consensus within the literature on pharmacists’ expertise in preventing, detecting, and managing these undesired effects in mental health settings [27–29]. The Society of Hospital Pharmacists Australia (SHPA) considers monitoring Adverse Drug Reactions (ADR) a core element of mental health pharmacy practice. It mandates psychiatric pharmacists to sustain a working knowledge on the incidence, prognosis, and treatment of psychotropic drug-related adverse events and perform routine screening, monitoring and reporting of ADRs [20].

The present study revealed that a slightly higher percentage of pharmacists (60%) evaluate patients for adherence to therapy, assess their information needs and engage in patient education and counselling. Compliance with therapy is one of the significant challenges to the stabilisation of patients with psychiatric illness. Therapeutic noncompliance has been associated with the incidence of therapeutic misadventures, prolonged admissions, relapse and subsequent readmissions [30–32]. A systematic review of the literature revealed that multi-faceted interventions involving patient education by pharmacists significantly improved patient adherence to therapy as well as their clinical outcomes [33]. This finding is consistent with the SHPA affirmation of the role of mental health pharmacists in patient education and counselling. Pharmacists are expected to employ effective counselling techniques, including providing verbal and written information to the patients or caregivers [20]. Our findings, however, indicate that most pharmacists did not provide the patients/ caretakers with educational or adherence supporting materials. Perhaps due to the lack of patient demand or the pharmacists’ inaccessibility to reliable information sources, both of which have been highly reported as barriers to the provision of pharmaceutical care in this study. This is a point of concern, particularly in view of role of supporting material in empowering patients, enhancing their knowledge and promoting their adherence to therapy [34–36].

The respondent’s level of education influenced the provision of professional pharmacy services, pharmacists with a bachelor`s degree educated and counselled patients at a higher frequency compared to pharmacists with other educational qualifications. This is contrary to findings from previous research that demonstrated an increased likelihood of provision of clinical pharmacy services, particularly among pharmacists with a graduate pharmacy degree [37]. Similarly, the practice setting had an impact on the services provided to patients. The evaluation of the appropriateness of the dosage form prescribed, the correctness of the drug/strength dispensed, the accuracy of patient medication list, the correctness of the drug administered, the use of expired medication and the potential for adverse drug reactions were performed to a greater extent by outpatient and ward pharmacists compared to other practice settings. Furthermore, they were more involved in the development and amendment of the patients’ pharmacotherapeutic plans along with the other healthcare professionals; this finding although unexpected is encouraged given the positive role of pharmacists in improving prescribing practices [17] and augmenting treatment outcomes, including enhanced care and levels of functioning at a reduced cost [38].

This study also found that pharmacists rarely work with patients and other healthcare professionals to design a patient-specific pharmacotherapeutic regimen and a corresponding monitoring plan. Formulating a patient-centred, outcomes-oriented pharmacotherapeutic plan to promote health, prevent disease and assure that drug therapy regimens are safe and effective is one of the core facets of pharmaceutical care. To do so, the pharmacists adopt a holistic approach to target the patient’s clinical conditions, incorporating the psycho-social aspects of the disease and the economic burden of both pharmacological and non-pharmacological treatments [39].

 Challenges across multiple levels influence the extent and scope of psychiatric pharmaceutical care services [40] including, individual, interpersonal, institutional, community and public policy-related factors. Individual factors, particularly the pharmacists’ knowledge/skills and confidence, have been corroborated by studies from Qatar, Kuwait, the United States and Malaysia as necessary for the successful expansion and implementation of pharmaceutical care [13, 22, 41, 42].

A large number of respondents agreed that patients have trouble understanding medical information; indeed, this has been emphasised in guidelines on the practice of mental health pharmacy [20]. Another interpersonal factor reported in our study and underlined in the literature is the pharmacists’ inability to communicate with the physicians. Effective communication skills and good rapport with physicians facilitate the extension of hospital pharmacy services [43].

Institutional factors perceived by the pharmacists and mirrored in studies across the globe include the lack of time [13, 22, 43–45], shortage of staff [13, 41, 42, 44, 46, 47] and inaccessibility to the patients’ medical records [13].

Likewise, the community-related factors highlighted by our research as well as the literature are the low patient demand and acceptance of pharmacy services [42, 48] and the lack of recognition from the physicians part to the pharmacists’ skills [47, 48].

Lastly, the lack of official policies and standardised practice protocols as observed in our study has also been cited in Qatar, Vietnam, and Western Pacific Countries [13, 47, 48].

This research has provided significant insight into the landscape of psychiatric pharmaceutical care within government hospitals in Malaysia. However, the anonymity of the survey process limited the exploration of the non-responders’ characteristics; hence, non-response bias cannot be eliminated. Moreover, the questionnaire was self-reported; hence there is potential for social desirability bias, i.e., the pharmacists responded positively to show support for the pharmaceutical care process. Furthermore, other data inaccuracies may have been introduced due to inherent recall bias. Nevertheless, the researchers attempted to minimise the potential for misinterpretation of the survey questions through face and content validation and piloting prior distribution. It is reasonable to assume that other barriers to psychiatric pharmaceutical care practice may have been overlooked, although the respondents were given the opportunity to free-write and add to the list. Finally, it seems plausible that the data only reflects the sampled pharmacists and not the nationwide practice due to the limitations in the sampling technique.

## Conclusion

This study has several potential implications for the practice of psychiatric pharmaceutical care in Malaysia. It has documented the scope and extent of the hospital pharmacy services provided to mental health patients and highlighted the need for mobilising pharmacists to expand these services in a convenience sample of government hospitals. Furthermore, it documented the impact of pharmacist education and the pharmacy setting on the level of pharmaceutical care services. The study also sheds light on several obstacles across different ecological levels of influence, including individual, interpersonal, institutional, community and public policy-related factors. The findings of this study can be used by the Ministry of Health and other relevant authorities to operationalise psychiatric pharmaceutical care in Malaysia. Although valuable information has been generated on expanding the pharmacists’ role in managing mental health conditions, further research is required to assess the impact of these services on patients within the Malaysian population.

## Data Availability

The datasets used and analysed during the current study are available from the corresponding author on reasonable request.

## Abbreviations

NCDs: Non-Communicable Diseases
DALYs: Disability-Adjusted Life Years
DRPs: Drug-Related Problems
PCNE: Pharmaceutical Care Network Europe
ASHP: American Society of Hospital Pharmacists
SHPA: Society of Hospital Pharmacists of Australia
SPSS: Statistical Package of Social Sciences
ADR: Adverse Drug Reactions
